# Supplementary material for: Deep-sea origin and depth colonization associated with phenotypic innovations in scleractinian corals
Source: Nat Commun. 2023 Nov 17;14:7458. doi: 10.1038/s41467-023-43287-y (PMC10656505; doi:10.1038/s41467-023-43287-y)
Supplement: Supplementary file 1 — Supplementary Information [file 41467_2023_43287_MOESM1_ESM.pdf]

Supplementary Information

Deep-sea origin and depth colonization associated with phenotypic innovations in scleractinian corals

Ana N. Campoy, Marcelo M. Rivadeneira, Cristián E. Hernández, Andrew Meade & Chris Venditti

Table of contents

Variation in the speed of colonization 1
    Supplementary Fig. 1 1
Preliminary analyses 2
    Supplementary Note 1 2
    Supplementary Fig. 2 2
Depth differences in corals with different traits 3
    Supplementary Fig. 3 3
    Supplementary Table 1 3
Evolution of depth differences in corals with different traits 4
    Supplementary Table 2 4
    Supplementary Table 3 4
    Supplementary Table 4 4
    Supplementary Table 5 5
    Supplementary Fig. 4 8
Diversification of scleractinian corals along the depth gradient 9
    Supplementary Table 6 9
    Supplementary Table 7 11
Lineage-specific evolutionary patterns in depth 12
    Supplementary Table 8 12
    Supplementary Table 9 12
    Supplementary Table 10 14
    Supplementary Table 11 14
    Supplementary Fig.5 15
Colonization rate in depth 16
    Supplementary Fig.6 16
Updated database 17
    Preliminary analyses 18
        Supplementary Note 2 18
        Supplementary Fig. 7 18
    Depth differences in corals with different traits 19
        Supplementary Fig. 8 19
        Supplementary Table 12 20
    Evolution of depth differences in corals with different traits 21
        Supplementary Table 13 21
        Supplementary Table 14 21
        Supplementary Table 15 22

Variation in the speed of colonization

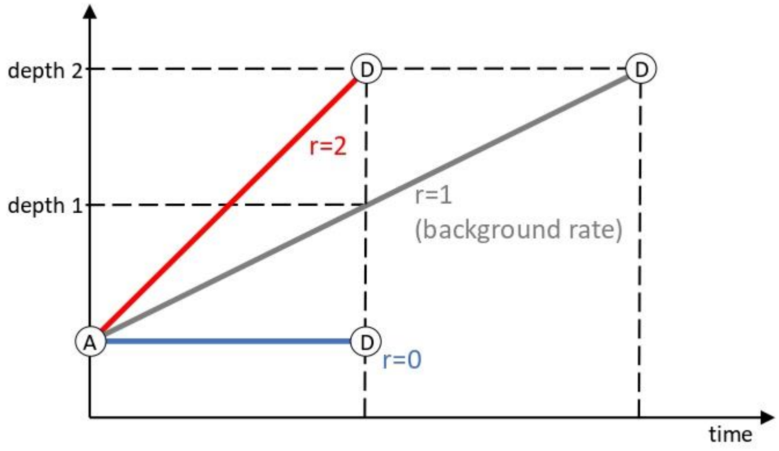

Supplementary Fig. 1 Schematisation of how a single lineage moving faster in the depth gradient (bigger lineage-specific scalar “r”) inherently exhibits a bigger colonization capacity. Lines represent three hypothetical lineages evolving at different rates from any ancestor (A) to its immediate descendant (D). r= lineage-specific scalar. A lineage moving at half velocity (e.g., the calculated background rate) must live twice as long to reach the same depth relative to its ancestor. A zero net change corresponds to a scalar of zero. Considering that the rate does not imply directionality, the change can occur toward shallower or deeper waters.

Preliminary analyses

Supplementary Note 1 The Spearman coefficient showed a significant correlation of the two datasets - *maximum depth reported* and *more conservative depth*- (n=510) for the three metrics (minimum: rho=0.83, S=3702400, p-value<2.2e-16; median: rho=0.87, S=2882700, p-value<2.2e-16; maximum: rho=0.88, S=2630200, p-value<2.2e-16). The branch scalars obtained from the variable rate model for each dataset were also correlated (median: rho=0.71, S=51180000, p-value<2.2e-16; maximum: rho=0.70, S=54232000, p-value<2.2e-16). This allowed us to exclude one dataset from further analyses, which were effectuated with the *more conservative depth* dataset.

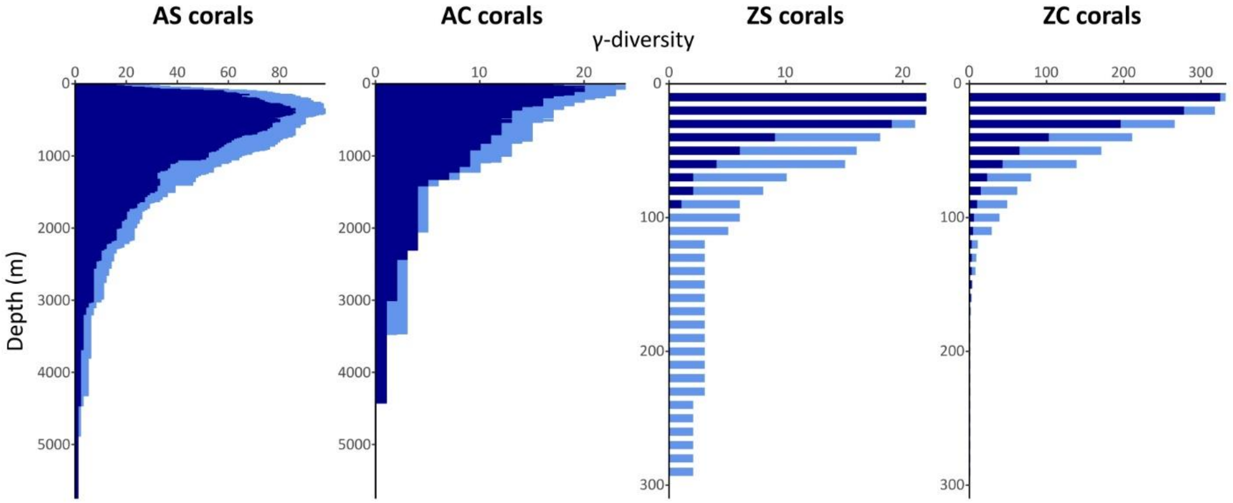

Supplementary Fig. 2 Pattern of bathymetric γ-diversity for the four scleractinian groups: AS (azooxanthellate solitary, n=108), AC (azooxanthellate colonial, n=31), ZS (zooxanthellate solitary, n=24) and ZC (zooxanthellate colonial, n=336). γ-diversity was calculated on 10 m bins using a range-through approach. The dark blue pattern is calculated from the conservative dataset, and the light blue from the maximum depth range reported for each species.

Depth differences in corals with different traits

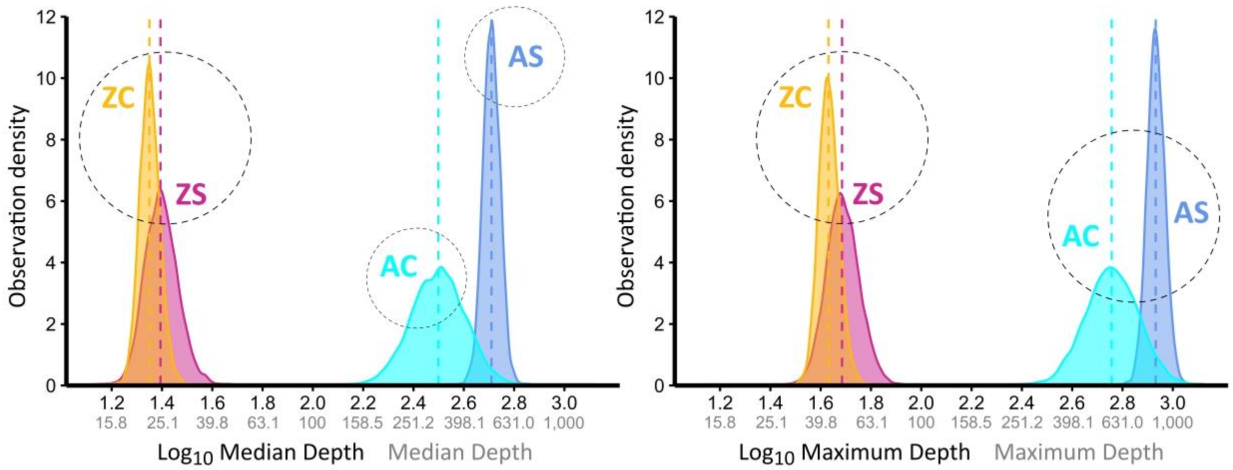

Supplementary Fig. 3 Phylogenetically corrected median and maximum $\log_{10}$ depth for each group of corals: AS (azooxanthellate solitary, n=108), AC (azooxanthellate colonial, n=31), ZS (zooxanthellate solitary, n=24) and ZC (zooxanthellate colonial, n=336), obtained from a variable rate phylogenetic regression model (Supplementary Table 1). Dashed lines point the phylogenetic median of the posterior distribution for the median and maximum depth (median depth: ZC=1.35 ~ 22.4 m, ZS=1.39 ~ 24.8 m, AC=2.50 ~ 316.2 m, AS=2.71 ~ 513.0 m; maximum depth: ZC=1.63 ~ 42.4 m, ZS=1.68 ~ 48.1 m, AC=2.75 ~ 567.5 m, AS=2.93 ~ 848.3 m). Dashed circles separate significantly different depths among groups. The significance was assessed as the proportion of the posterior distribution in which differences in estimated coefficients cross zero using a threshold value of 0.05 ($p < 0.05$), i.e., median depth: $p_{AS\text{-}AC}=0.02$, $p_{AS\text{-}ZS}<0.00$, $p_{AS\text{-}ZC}<0.00$, $p_{AC\text{-}ZS}<0.00$, $p_{AC\text{-}ZC}<0.00$, $p_{ZS\text{-}ZC}=0.18$; maximum depth: $p_{AS\text{-}AC}=0.05$, $p_{AS\text{-}ZS}<0.00$, $p_{AS\text{-}ZC}<0.00$, $p_{AC\text{-}ZS}<0.00$, $p_{AC\text{-}ZC}<0.00$, $p_{ZS\text{-}ZC}=0.14$.

Supplementary Table 1 Brownian motion (BM) and variable rate (VR) models of depth evolution for the four groups of scleractinian corals: AS (azooxanthellate solitary, n=108), AC (azooxanthellate colonial, n=31), ZS (zooxanthellate solitary, n=24) and ZC (zooxanthellate colonial, n=336). Log m Lh: log marginal likelihood. λ: inferred phylogenetic signal - median of the posterior. Log Bayes Factor (BF) higher than 10 indicates very strong evidence in favour of the VR model.

		BM model		VR model		Log BF
		**Log m Lh**	**λ**	**Log m Lh**	**λ**	
**Maximum depth** ($\log_{10}$ max. depth)	Run 01	-107.28	0.37	-73.27	0.45	68.01
	Run 02	-107.34	0.37	-73.13	0.45	68.42
	Run 03	-107.35	0.37	-73.01	0.45	68.66
**Median depth** ($\log_{10}$ med. depth)	Run 01	-93.04	0.39	-52.32	0.47	81.44
	Run 02	-92.86	0.39	-52.71	0.46	80.30
	Run 03	-92.89	0.39	-52.83	0.49	80.13

*$\log_{10}$ depth ~ $\alpha^{AS}+\beta_1^{AC}+\beta_2^{ZS}+\beta_3^{ZC}$

Evolution of depth differences in corals with different traits

Supplementary Table 2 Brownian motion (BM) and variable rate (VR) models of depth evolution for scleractinian corals (n=510). Log m Lh: log marginal likelihood. λ: inferred phylogenetic signal - median of the posterior. Log Bayes Factors (BF) higher than 10 indicates very strong evidence in favour of the VR model.

		BM model		VR model		Log BF
		Log m Lh	λ	Log m Lh	λ	
**Maximum depth** (log_{10} max. depth)	Run 01	-222.14	0.85	-159.23	0.90	125.83
	Run 02	-222.11	0.85	-169.32	0.91	105.58
	Run 03	-222.21	0.85	-151.74	0.91	140.93
**Median depth** (log_{10} med. depth)	Run 01	-214.80	0.86	-137.13	0.90	155.34
	Run 02	-214.79	0.86	-154.08	0.89	121.42
	Run 03	-214.74	0.86	-140.78	0.90	147.90

Supplementary Table 3 Brownian motion (BM) and variable rate (VR) models of depth evolution, including latitude extension, accounting for differences among four groups of scleractinian corals: AS (azooxanthellate solitary, n=108), AC (azooxanthellate colonial, n=31), ZS (zooxanthellate solitary, n=24) and ZC (zooxanthellate colonial, n=336). Facultative species (n=11) are also included, coded as 0.5 for the two groups they are part of. Log m Lh: log marginal likelihood. λ: inferred phylogenetic signal - median of the posterior. R^2: coefficient of determination - median of the posterior. Log Bayes Factor (BF) higher than 10 indicates very strong evidence in favour of the VR model.

		BM model			VR model			Log BF
		Log m Lh	λ	R^2	Log m Lh	λ	R^2	
**Maximum depth** (log_{10} max. depth)	Run 01	-67.37	0.27	0.56	-67.37	0.35	0.85	100.7
	Run 02	-67.36	0.26	0.56	-67.36	0.36	0.69	103.01
	Run 03	-67.67	0.27	0.56	-67.67	0.36	0.69	100.52
**Median depth** (log_{10} med. depth)	Run 01	-136.97	0.41	0.46	-83.97	0.39	0.64	105.99
	Run 02	-136.13	0.41	0.46	-86.52	0.38	0.64	99.22
	Run 03	-135.94	0.42	0.46	-84.20	0.44	0.64	103.49

Log_{10} depth ~ $\alpha^{AS}+\beta_1^{AS}$(latitude)+$\beta_2^{AC}+\beta_3^{AC}$*(latitude)+$\beta_4^{ZS}+\beta_5^{ZS}$*(latitude)+$\beta_6^{ZC}+\beta_7^{ZC}$*(latitude)

Supplementary Table 4 Comparison of the model of depth evolution (Supplementary Table 2) and the same model after accounting for the effect of latitudinal extension (Supplementary Table 3). Log m Lh: log marginal likelihood. Log Bayes Factors (BF) higher than 10 indicates very strong evidence in favour of the complex model.

		Log m Lh		Log BF
		Depth	Depth ~ Latitude	
**Maximum depth**	Run 01	-159.23	-67.37	183.72
	Run 02	-169.32	-67.36	203.92
	Run 03	-151.74	-67.67	168.14
**Median depth**	Run 01	-137.13	-83.97	106.32
	Run 02	-154.08	-86.52	135.12
	Run 03	-140.78	-84.20	113.16

Supplementary Table 5 Regression model of depth evolution including latitude (Supplementary Table 3) and progressive reduction accounting for differences among four groups of scleractinian corals: AS (azooxanthellate solitary, n=108), AC (azooxanthellate colonial, n=31), ZS (zooxanthellate solitary, n=24) and ZC (zooxanthellate colonial, n=336). Facultative species (n=11) are also included, coded as 0.5 for the two groups they are part of. N=510. Regression parameters in red were non-significant (p<0.05). Maximum depth: full model - p<0.00 except for $p(\beta_5^{ZS})=0.17$; reduction 1 – p<0.00 except for $p(\beta_4^{ZS})=0.30$; reduction 2 – p<0.00 except for $p(\beta_4^{ZS})=0.31$. Median depth: full model - p<0.00 except for $p(\beta_1^{AS})=0.01$, $p(\beta_3^{AC})=0.02$, $p(\beta_5^{ZS})=0.13$ and $p(\beta_7^{ZC})=0.11$; reduction 1 – p<0.00 except for $p(\beta_3^{AC})=0.01$ and $p(\beta_6^{ZC})=0.13$; reduction 2 – p<0.00 except for $p(\beta_5^{ZC})=0.09$; reduction 3 – p<0.00. Log m Lh: log marginal likelihood. λ: inferred phylogenetic signal - median of the posterior. R^2: coefficient of determination - median of the posterior. One parameter was eliminated at a time, joining in the next run those intercepts or slopes for which the proportion of their difference was bigger and outermost from 0.5. Maximum depth: full model – intercepts β_2-β_4 (p=0.15); reduction 1 – slopes β_1-β_6 (p=0.07). Median depth: full model – slopes β_1-β_5 (p=0.47); reduction 1 – intercepts β_4-β_5 (p=0.48); reduction 2 – slopes β_1-β_5 (p=0.05). Each reduction was compared with the previous model. Log Bayes Factors (BF) of 5-10 indicates strong evidence in favour of the reduced model, while BF higher than 10 shows very strong evidence.

Maximum depth

FULL MODEL – 4 intercepts, 4 slopes

Log_{10} depth (max.) ~ $\alpha^{AS}+\beta_1^{AS}*(\text{latitude})+\beta_2^{AC}+\beta_3^{AC}*(\text{latitude})+\beta_4^{ZS}+\beta_5^{ZS}*(\text{latitude})+\beta_6^{ZC}+\beta_7^{ZC}*(\text{latitude})$

Log_{10} depth (max.) ~ 2.42+0.01*latitude+1.82+0.02*latitude+1.55+0.00*latitude+1.30+0.01*latitude

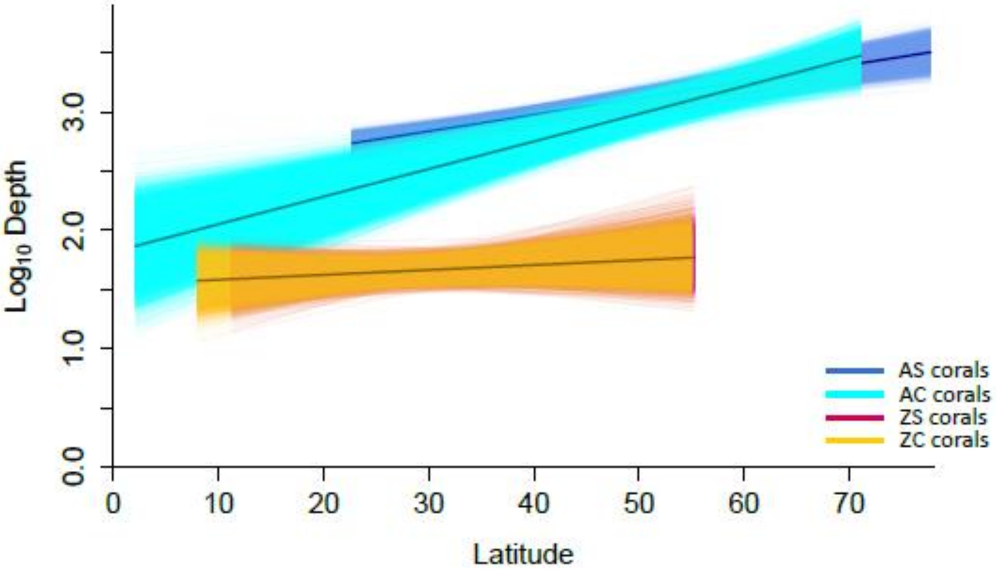

	Log m Lh	λ	R^2	Log BF
Run 01	-67.37	0.35	0.85	-
Run 02	-67.36	0.36	0.69	-
Run 03	-67.67	0.36	0.69	-

REDUCTION 1 – 3 intercepts, 4 slopes

Log_{10} depth (max.) ~ $\alpha^{AS}+\beta_1^{AS}*(\text{latitude})+\beta_2^{AC,ZS}+\beta_3^{AC}*(\text{latitude})+\beta_4^{ZS}*(\text{latitude})+\beta_5^{ZC}+\beta_6^{ZC}*(\text{latitude})$

Log_{10} depth (max.) ~ 2.41+0.01*latitude+1.62+0.03*latitude+0.02*latitude+1.31+0.01*latitude

Run 01	-62.10	0.32	0.71	10.53
Run 02	-61.69	0.33	0.70	11.35
Run 03	-63.69	0.34	0.70	7.96

REDUCTION 2 – 3 intercepts, 3 slopes

Log_{10} depth (max.) ~ $\alpha^{AS}+\beta_1^{AS,ZC}*(\text{latitude})+\beta_2^{AC,ZS}+\beta_3^{AC}*(\text{latitude})+\beta_4^{ZS}*(\text{latitude})+\beta_5^{ZC}$

Log_{10} depth (max.) ~ 2.47+0.01*latitude+1.61+0.03*latitude+0.00*latitude+1.24

Run 01	-52.42	0.36	0.69	19.36
Run 02	-52.26	0.35	0.69	18.86
Run 03	-54.16	0.36	0.69	19.06

Median depth

FULL MODEL – 4 intercepts, 4 slopes

Log_{10} depth (med.) ~ $\alpha^{AS}+\beta_1^{AS}*(\text{latitude})+\beta_2^{AC}+\beta_3^{AC}*(\text{latitude})+\beta_4^{ZS}+\beta_5^{ZS}*(\text{latitude})+\beta_6^{ZC}+\beta_7^{ZC}*(\text{latitude})$

Log_{10} depth (med.) ~ 2.55+0.01*latitude+1.95+0.03*latitude+1.26+0.01*latitude+1.28+0.00*latitude

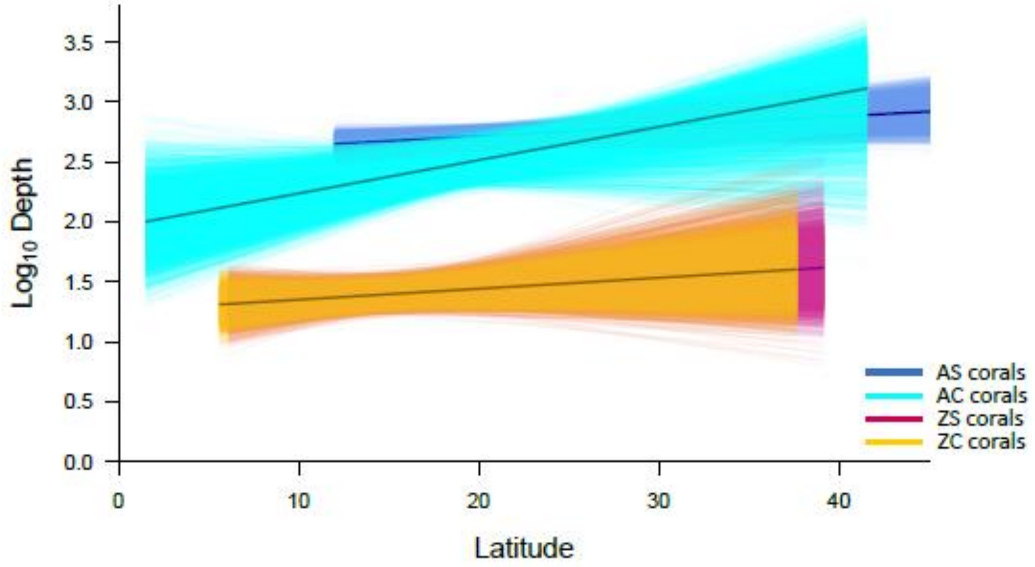

	Log m Lh	λ	R^2	Log BF
Run 01	-83.97	0.39	0.64	-
Run 02	-86.52	0.38	0.64	-
Run 03	-84.20	0.44	0.64	-

REDUCTION 1 – 4 intercepts, 3 slopes

Log_{10} depth (med.) ~ $\alpha^{AS}+\beta_1^{AS,ZS}*(\text{latitude})+\beta_2^{AC}+\beta_3^{AC}*(\text{latitude})+\beta_4^{ZS}+\beta_5^{ZC}+\beta_6^{ZC}*(\text{latitude})$

Log_{10} depth (med.) ~ 2.56+0.01*latitude+1.94+0.03*latitude+1.27+1.28+0.00*latitude

	Log m Lh	λ	R^2	Log BF
Run 01	-76.23	0.42	0.64	15.48
Run 02	-75.93	0.23	0.94	21.29
Run 03	-75.47	0.43	0.64	17.45

REDUCTION 2 – 3 intercepts, 3 slopes

Log_{10} depth (med.) ~ $\alpha^{AS}+\beta_1^{AS,ZS}*(\text{latitude})+\beta_2^{AC}+\beta_3^{AC}*(\text{latitude})+\beta_4^{ZS,ZC}+\beta_5^{ZC}*(\text{latitude})$

Log_{10} depth (med.) ~ 2.56+0.01*latitude+1.94+0.03*latitude+1.28+0.00*latitude

	Log m Lh	λ	R^2	Log BF
Run 01	-66.77	0.45	0.64	18.92
Run 02	-68.89	0.40	0.64	14.08
Run 03	-67.73	0.43	0.64	15.49

REDUCTION 3 – 3 intercepts, 2 slopes

Log_{10} depth (med.) ~ $\alpha^{AS}+\beta_1^{AS,ZS,ZC}*(\text{latitude})+\beta_2^{AC}+\beta_3^{AC}*(\text{latitude})+\beta_4^{ZS,ZC}$

Log_{10} depth (med.) ~ 2.60+0.01*latitude+1.95+0.03*latitude+1.24

	Log m Lh	λ	R^2	Log BF
Run 01	-59.48	0.42	0.64	14.58
Run 02	-60.00	0.42	0.64	17.78
Run 03	-60.77	0.42	0.64	13.92

Supplementary Fig. 4 Median trees obtained from the best models of depth evolution accounting for differences among the four groups of scleractinian corals: AS (azooxanthellate solitary, n=108), AC (azooxanthellate colonial, n=31), ZS (zooxanthellate solitary, n=24) and ZC (zooxanthellate colonial, n=336). Facultative species (n=11) are also included, coded as 0.5 for the two groups they are part of. Branches are modified according to the rate of evolution (r). Tree branches' colour and length reflect shifts in the rate of depth evolution. Grey branches are lineages of constant depth evolution (background rate, r=1), blue branches (r<1) are compressed and reflect slower rates, while red branches reflect accelerated rates (r>1). The traditional names of each clade are presented on the right side to enhance the visualization of the results.

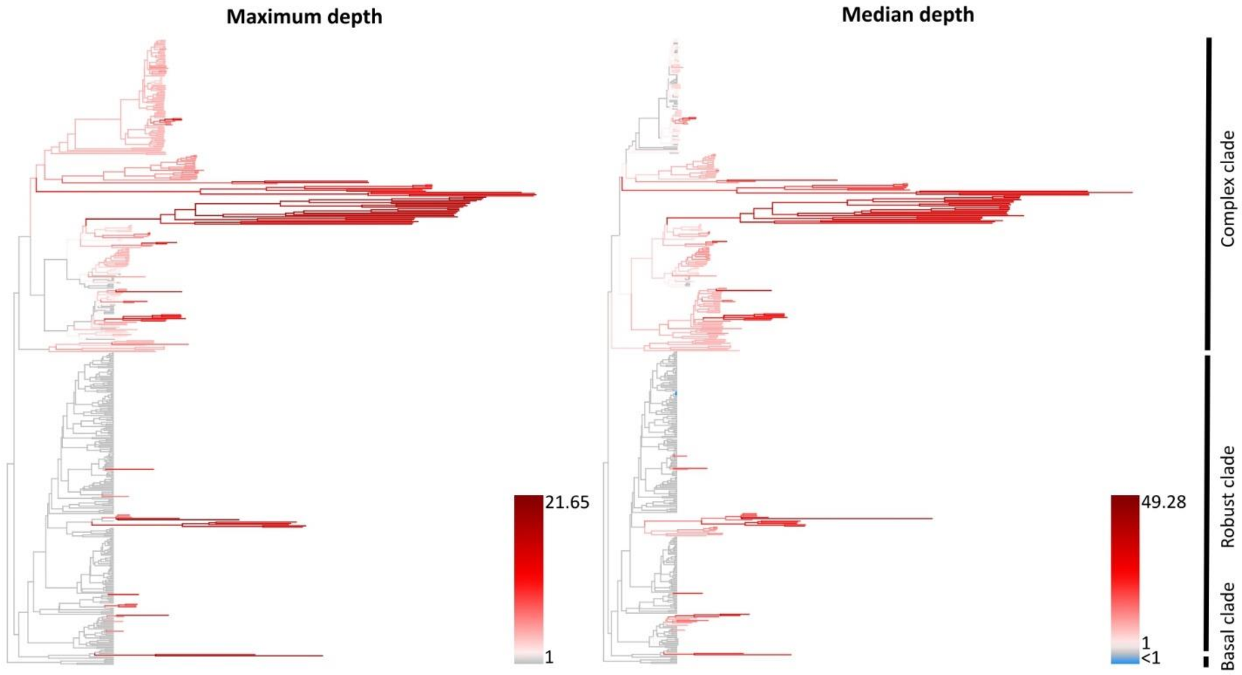

Log_{10} depth (max.) ~ $2.47^{AS}+0.01^{AS,ZC}*(\text{latitude})+1.61^{AC,ZS}+0.03^{AC}*(\text{latitude})+0.00^{ZS}*(\text{latitude})+1.24^{ZC}$

Log_{10} depth (med.) ~ $2.60^{AS}+0.01^{AS,ZS,ZC}*(\text{latitude})+1.95^{AC}+0.03^{AC}*(\text{latitude})+1.24^{ZS,ZC}$

Diversification of scleractinian corals along the depth gradient

Supplementary Table 6 Depth trends in the path-wise rate or distance from the root to the tips accounting for differences among the four groups of scleractinian corals: AS (azooxanthellate solitary, n=108), AC (azooxanthellate colonial, n=31), ZS (zooxanthellate solitary, n=24) and ZC (zooxanthellate colonial, n=336). Facultative species (n=11) are also included, coded as 0.5 for the two groups they are part of. N=510. Log m Lh: log marginal likelihood. λ: inferred phylogenetic signal - median of the posterior. R^2: coefficient of determination - median of the posterior. Non-significant regression parameters are highlighted in red (p<0.05). Maximum depth: p<0.00 except for $p(\beta_5^{ZS})=0.48$ and $p(\beta_7^{ZC})=0.39$. Median depth: p<0.00 except for $p(\beta_5^{ZS})=0.46$ and $p(\beta_7^{ZC})=0.33$.

Maximum depth

Log_{10} depth (max.) ~ $\alpha^{AS}+\beta_1^{AS}$*(path-wise rate)+$\beta_2^{AC}+\beta_3^{AC}$*(path-wise rate)+$\beta_4^{ZS}+\beta_5^{ZS}$*(path-wise rate) +$\beta_6^{ZC}+\beta_7^{ZC}$*(path-wise rate)

Log_{10} depth (max.) ~ 3.17-0.00*(path-wise rate) +3.04-0.00*(path-wise rate) +1.68+0.00*(path-wise rate) +1.62-0.00*(path-wise rate)

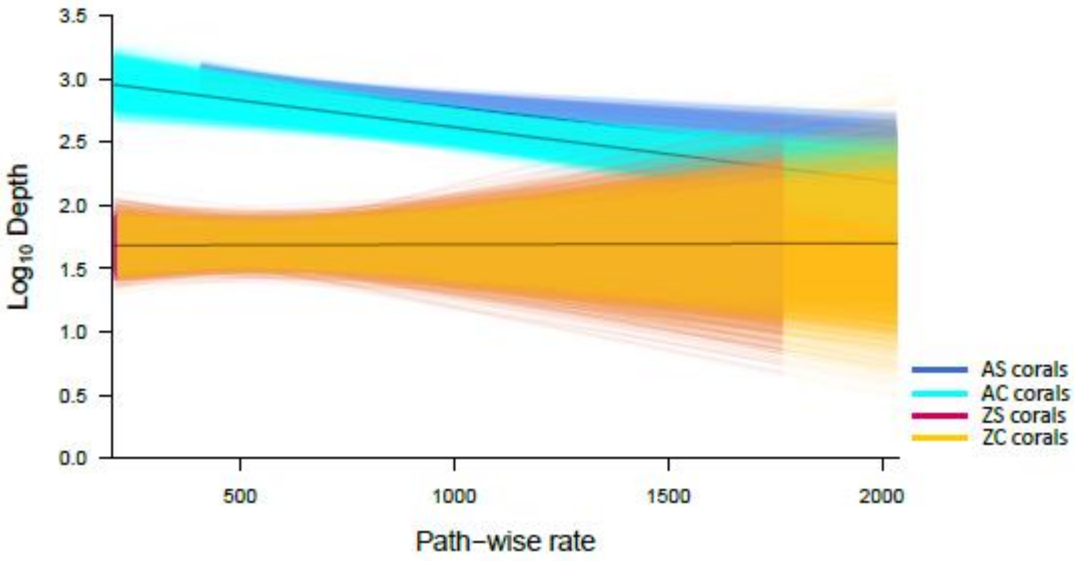

	Log m Lh	λ	R^2
Run 01	-152.77	0.24	0.54
Run 02	-152.39	0.24	0.54
Run 03	-152.95	0.24	0.54

Median depth

Log_{10} depth (med.) ~ $\alpha^{AS}+\beta_1^{AS}$*(path-wise rate)+$\beta_2^{AC}+\beta_3^{AC}$*(path-wise rate)+$\beta_4^{ZS}+\beta_5^{ZS}$*(path-wise rate) +$\beta_6^{ZC}+\beta_7^{ZC}$*(path-wise rate)

Log_{10} depth (med.) ~ 2.91-0.00*(path-wise rate) +2.82-0.00*(path-wise rate) +1.39-0.00*(path-wise rate) +1.34-0.00*(path-wise rate)

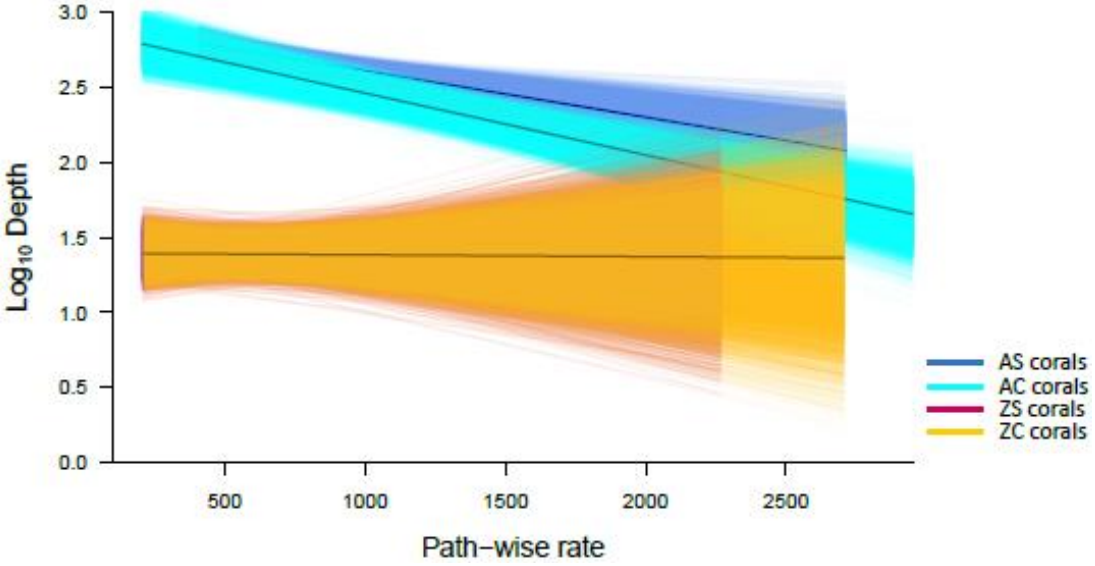

	Log m Lh	λ	R^2
Run 01	-128.40	0.23	0.58
Run 02	-129.03	0.23	0.58
Run 03	-128.44	0.23	0.58

Supplementary Table 7 Parameters of the Generalized Least Squares model of depth evolution to predict depth values at the internal nodes of the tree while accounting for differences among the four groups of scleractinian corals: AS (azooxanthellate solitary, n=108), AC (azooxanthellate colonial, n=31), ZS (zooxanthellate solitary, n=24) and ZC (zooxanthellate colonial, n=336). Facultative species (n=11) are also included, coded as 0.5 for the two groups they are part of. N=510. Lambda statistic=1. Non-significant regression parameters are highlighted in red (p<0.05).

Maximum depth

Log_{10} depth (max.) ~ $\alpha^{AS}+\beta_1^{AS}*$(path-wise rate)$+\beta_2^{AC}+\beta_3^{AC}*$(path-wise rate)$+\beta_4^{ZS,ZC}+\beta_5^{ZS,ZC}*$(path-wise rate)

Maximized log-likelihood= -108.97 / AIC= 234.98 / AICc= 235.15

Null Mean Square= 0.70, Residual Mean Square= 0.55, Raw R^2= 0.21, Adjusted R^2= 0.20
F statistic= 26.78, P model= 0

SUMMARY OF COEFFICIENTS

	Estimate	Std Error	T-value	P
(INTERCEPT)	3.36	0.31	10.83	0
PATH-WISE (AS)	-0.00	0.00	-2.21	0.03
AC	-0.32	0.16	-2.05	0.04
ZSZC	-1.49	0.22	-6.71	0.00
PATH-WISE (AS):AC	0.00	0.00	0.66	0.51
PATH-WISE (AS):ZSZC	0.00	0.00	1.26	0.21

Median depth

Log_{10} depth (med.) ~ $\alpha^{AS}+\beta_1^{AS}*$(path-wise rate)$+\beta_2^{AC}+\beta_3^{AC}*$(path-wise rate)$+\beta_4^{ZS,ZC}+\beta_5^{ZS,ZC}*$(path-wise rate)

Maximized log-likelihood= -79.73 / AIC= 176.49 / AICc= 176.66

Null Mean Square= 0.65, Residual Mean Square= 0.48, Raw R^2= 0.27, Adjusted R^2= 0.26
F statistic=36.87, P model= 0

SUMMARY OF COEFFICIENTS

	Estimate	Std Error	T-value	P
(INTERCEPT)	3.10	0.28	10.99	0
PATH-WISE (AS)	-0.00	0.00	-2.40	0.02
AC	-0.31	0.14	-2.30	0.02
ZSZC	-1.45	0.19	-7.52	0.00
PATH-WISE (AS):AC	0.00	0.00	0.83	0.41
PATH-WISE (AS):ZSZC	0.00	0.00	0.85	0.40

Lineage-specific evolutionary patterns in depth

A. Depth colonization rate

Supplementary Table 8 Diagonal elements are median rate values obtained using a local transformation on the variable rate model for each trait-defined lineage partition (n=1,018) across a sample of 500 trees. Rate values have a comparative meaning among partitions. Rates were standardised and log-transformed to test for differences using a Kruskal Wallis test with a Holm correction on the p-values. Elements in the lower triangle of the table are pairwise comparisons (significant values at p<0.05). Non-significant comparisons are in red colour. Groups are AC: azooxanthellate colonial, ZC: zooxanthellate colonial, UncS: uncertain for symbiosis, TransC: transition for coloniality, AS: azooxanthellate solitary, ZS: zooxanthellate solitary, UncC: uncertain for coloniality and TransS: transition for symbiosis.

	AC	ZC	UncS	TransC	AS	ZS	UncC	TransS
AC	**0.901**	-	-	-	-	-	-	-
ZC	0.000	**0.615**	-	-	-	-	-	-
UncS	0.000	0.345	**0.606**	-	-	-	-	-
TransC	0.000	0.000	0.045	**0.543**	-	-	-	-
AS	0.000	0.000	0.002	0.429	**0.531**	-	-	-
ZS	0.000	0.002	0.000	0.003	0.004	**0.497**	-	-
UncC	0.000	0.000	0.000	0.000	0.000	0.000	**0.354**	-
TransS	0.000	0.000	0.000	0.000	0.000	0.000	0.000	**0.156**

B. Changes in lineage depth ranges

Supplementary Table 9 Phylogenetic ancestor-descendant (PAD) comparisons. Total number of branches and branches exhibiting changes toward shallower waters in the minimum and maximum depth, and changes in the median depth occurring at a background/decelerated (r≤1) or accelerated colonization rate (r>1). The predominance for shallow changes is evaluated using a two-sided binomial test. Non-significant changes are highlighted in red (p<0.05). Groups are AS: azooxanthellate solitary, AC: azooxanthellate colonial, ZS: zooxanthellate solitary, ZC: zooxanthellate colonial, TransS: transition for symbiosis, TransC: transition for coloniality, UncS: uncertain for symbiosis and UncC: uncertain for coloniality.

Group	Depth	Rate	Total	Shallow	% Shallow	p
All	min. depth		1018	652	64.05	<0.001
	max. depth			612	60.12	<0.001
	med. depth	r≤1	500	309	61.80	<0.001
		r>1	518	308	59.46	<0.001

AS	min. depth		195	138	70.77	<0.001
	max. depth			126	64.62	<0.001
	med. depth	r≤1	53	40	75.47	0.000
		r>1	142	90	63.38	0.002
AC	min. depth		38	33	86.84	<0.001
	max. depth			24	63.16	0.143
	med. depth	r≤1	3	0	0	0.250
		r>1	35	24	68.57	0.041
ZS	min. depth		52	46	88.46	<0.001
	max. depth			36	69.23	0.008
	med. depth	r≤1	51	37	72.55	0.002
		r>1	1	0	0	1
ZC	min. depth		610	441	72.30	<0.001
	max. depth			339	55.57	0.007
	med. depth	r≤1	331	194	58.61	0.002
		r>1	279	150	53.76	0.231
TransS	min. depth		23	23	100	<0.001
	max. depth			23	100	<0.001
	med. depth	r≤1	10	10	100	0.002
		r>1	13	13	100	0.000
TransC	min. depth		16	11	68.75	0.21
	max. depth			8	50	1
	med. depth	r≤1	11	6	54.55	1
		r>1	5	2	40	1
UncS	min. depth		7	4	57.14	1
	max. depth			3	42.86	1
	med. depth	r≤1	1	0	0	1
		r>1	6	3	50	1
UncC	min. depth		77	48	62.34	0.04
	max. depth			53	68.83	0.001
	med. depth	r≤1	40	26	65	0.081
		r>1	37	26	70.27	0.02

Supplementary Table 10 Average (± standard deviation) depth changes for each trait-defined lineage using the PAD comparisons approach (difference descendant-ancestor in the median depth). Groups are AS: azooxanthellate solitary, AC: azooxanthellate colonial, ZS: zooxanthellate solitary, ZC: zooxanthellate colonial, TransS: transition for symbiosis, TransC: transition for coloniality, UncS: uncertain for symbiosis and UncC: uncertain for coloniality.

Group	Log_{10} depth change	% Depth change
AS	-0.07 ±0.21	2.50
AC	-0.19 ±0.40	7.64
ZS	-0.00 ±0.08	0.02
ZC	-0.04 ±0.13	2.98
TransS	-1.19 ±0.14	44.26
TransC	-0.04 ±0.22	2.16
UncS	0.17 ±0.57	8.48
UncC	-0.09 ±0.21	3.48

C. Long-term trends

Supplementary Table 11 Number of nodes and tips (N.Branches=1,018) originated from each branch state (defined by the two traits: symbiosis and coloniality) that evolved at constant (BM) or decelerated rates (r≤1, n=496) and accelerated rates (r>1, n=506). Result obtained from the best model of depth evolution (Supplementary Fig. 4). Black cells identify groups excluded in subsequent regression models (Supplementary Fig. 5) for not having enough representants (criteria: n< 10). The partition TransS_UncC was merged with TransS as the evolutionary rates were similar, and each group independently had insufficient representatives.

Symbiosis	Coloniality	N.Branches	Partition	r≤1	r>1
azooxanthellate	solitary	195	**AS**	53 (27.2%)	142 (72.8%)
azooxanthellate	colonial	38	**AC**	3 (7.9%)	35 (92.1%)
zooxanthellate	solitary	52	**ZS**	51 (98.1%)	1 (1.9%)
zooxanthellate	colonial	610	**ZC**	331 (54.3%)	279 (45.7%)
transition	solitary	1	**TransS**	10 (43.5%)	13 (56.5%)
	colonial	9			
	uncertain	13			
uncertain	colonial	6	**UncS**	1 (14.3%)	6 (85.7%)
	uncertain	1			
azooxanthellate	transition	4	**TransC**	11 (68.8%)	5 (31.3%)
zooxanzellate		12			
azooxanthellate	uncertain	64	**UncC**	40 (51.9%)	37 (48.1%)
zooxanthellate		13			
				**500 (496)**	**518 (506)**

Supplementary Fig. 5 Bayesian multiple regression models to test trends in depth over time for species originated from lineages of background/slow (r≤1, n=496) and fast colonization rate (r>1, n=506). Trait-defined lineages with n<10 were excluded (see Supplementary Table 11). Non-significant regression parameters are highlighted in red (p<0.05). Background/slow colonization: p<0.00 except for the slopes of ZS (p=0.27), TransS (p=0.15) and TransC (p=0.08). Fast colonization: p<0.00 except for the slopes of ZC (p=0.02) and TransS (p=0.37). AS: azooxanthellate solitary, AC: azooxanthellate colonial, ZS: zooxanthellate solitary, ZC: zooxanthellate colonial, TransS: transition for symbiosis, TransC: transition for coloniality, UncC: uncertain for coloniality.

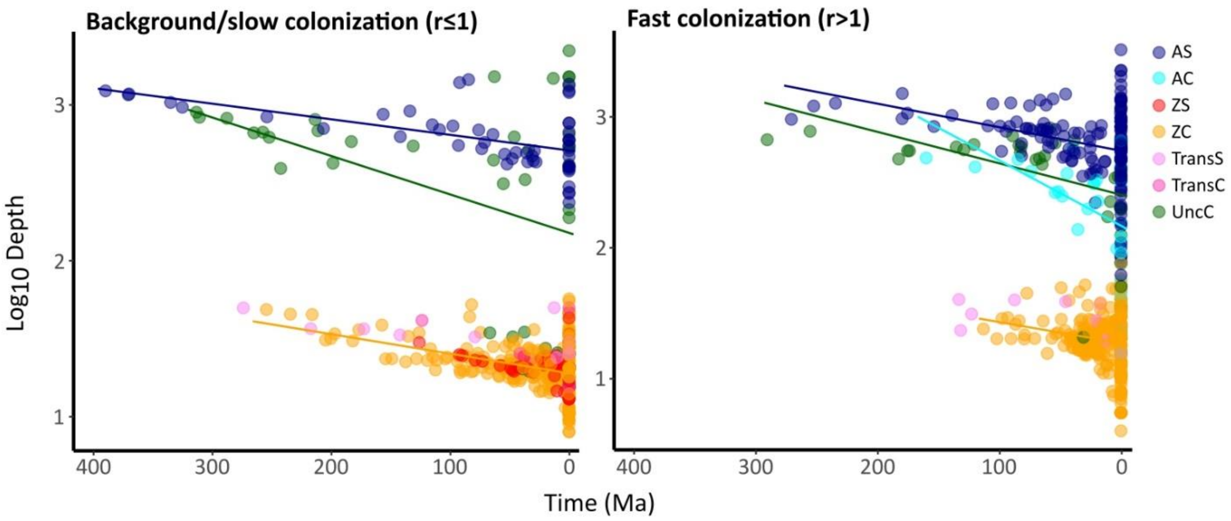

Background/slow colonization rate (r≤1): $\log_{10}$ depth (med.) ~ 3.12^{AS} -0.00^{AS}(time) $+1.59^{ZS}$ -0.00^{ZS}*(time) $+1.79^{ZC}$ -0.00^{ZC}*(time) $+1.76^{TransS}$ -0.00^{TransS}*(time) $+2.33^{TransC}$ -0.00^{TransC}*(time) $+3.21^{UncC}$ -0.00^{UncC}*(time). R^2=0.85

Fast colonization rate (r>1): $\log_{10}$ depth (med.) ~ 3.49^{AS} -0.00^{AS}(time)$+4.25^{AC}$ -0.01^{AC}*(time) $+1.94^{ZC}$ -0.00^{ZC}*(time)$+1.67^{TransS}$ -0.00^{TransS}*(time) $+3.40^{UncC}$ -0.00^{UncC}*(time). R^2=0.87

Colonization rate in depth

Supplementary Fig. 6 Bayesian multiple regression model to test if deeper species originated at a slower colonization rate (n=843). Non-significant regression parameters are highlighted in red (p<0.05). p<0.00 except for the slope of ZC (p=0.19). AS: azooxanthellate solitary, AC: azooxanthellate colonial, ZC: zooxanthellate colonial. ZS (zooxanthellate solitary) were excluded (n<10, see Supplementary Table 11).

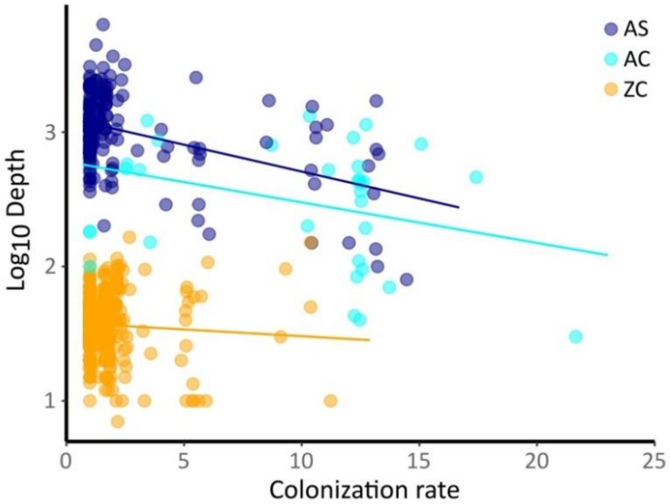

Log_{10} depth (max.) ~ 3.11^{AS} -0.04^{AS}(scalar) $+2.78^{AC}$ -0.03^{AC}*(scalar) $+1.58^{ZC}$ -0.01^{ZC}*(scalar). R^2=0.89

Updated database

The database utilized in this study was updated in December 2022, and subsequent analyses were conducted using this updated version. Statistical values and figures obtained with the older database (previous section of this file) are also included here for the purpose of comparing the results obtained from the two databases. Only sample sizes and statistics obtained with the older database that differed from those obtained with the updated databased are shown enclosed in parentheses (blue colour).

Preliminary analyses

Supplementary Note 2 The Spearman coefficient showed a significant correlation of the two datasets - *maximum depth reported* and *more conservative depth* - (n=509 (n=510)) for the three metrics (minimum: rho=0.79 (0.83), S=4544052 (3702400), p-value<2.2e-16; median: rho=0.89 (0.87), S=2327811 (2882700), p-value<2.2e-16; maximum: rho=0.90 (0.88), S=2230449 (2630200), p-value<2.2e-16). The branch scalars obtained from the variable rate model for each dataset were also correlated (median: rho=0.81 (0.71), S=33582070 (51180000), p-value<2.2e-16; maximum: rho=0.77 (0.70), S=40276885 (542320000), p-value<2.2e-16). This allowed us to exclude one dataset from further analyses, which were effectuated with the *more conservative depth* dataset.

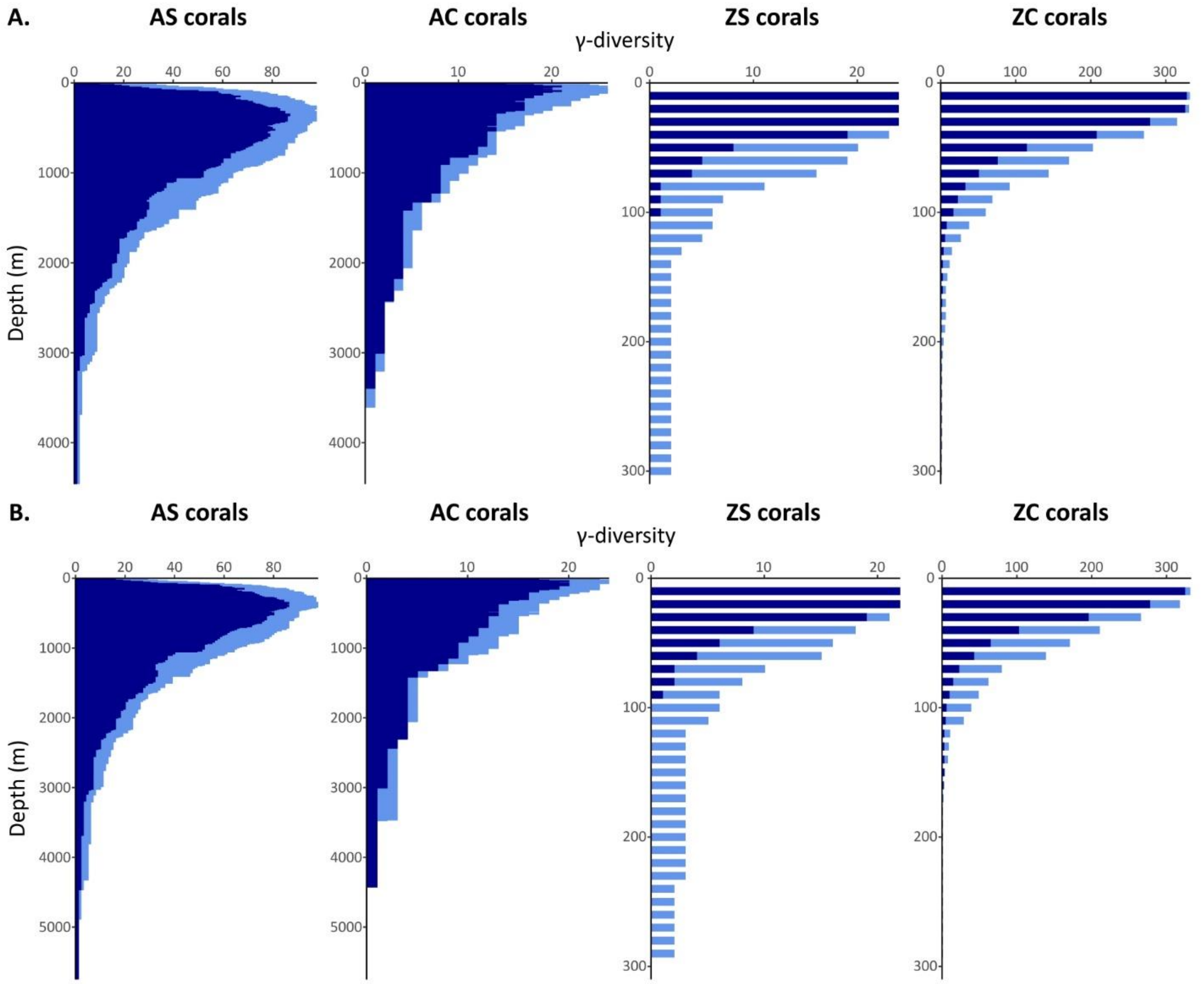

Supplementary Fig. 7 Pattern of bathymetric γ-diversity for four groups of scleractinian corals: AS (azooxanthellate solitary, n_A=107 (n_B=108)), AC (azooxanthellate colonial, $n_{A,B}$=31), ZS (zooxanthellate solitary, $n_{A,B}$=24), ZC (zooxanthellate colonial, $n_{A,B}$=336). γ-diversity was calculated on 10 m bins using a range-through approach. The dark blue pattern is calculated from the conservative dataset, and the light blue from the maximum depth range reported for each species. A. Updated database. B. Older database (differences with A. in blue colour).

Depth differences in corals with different traits

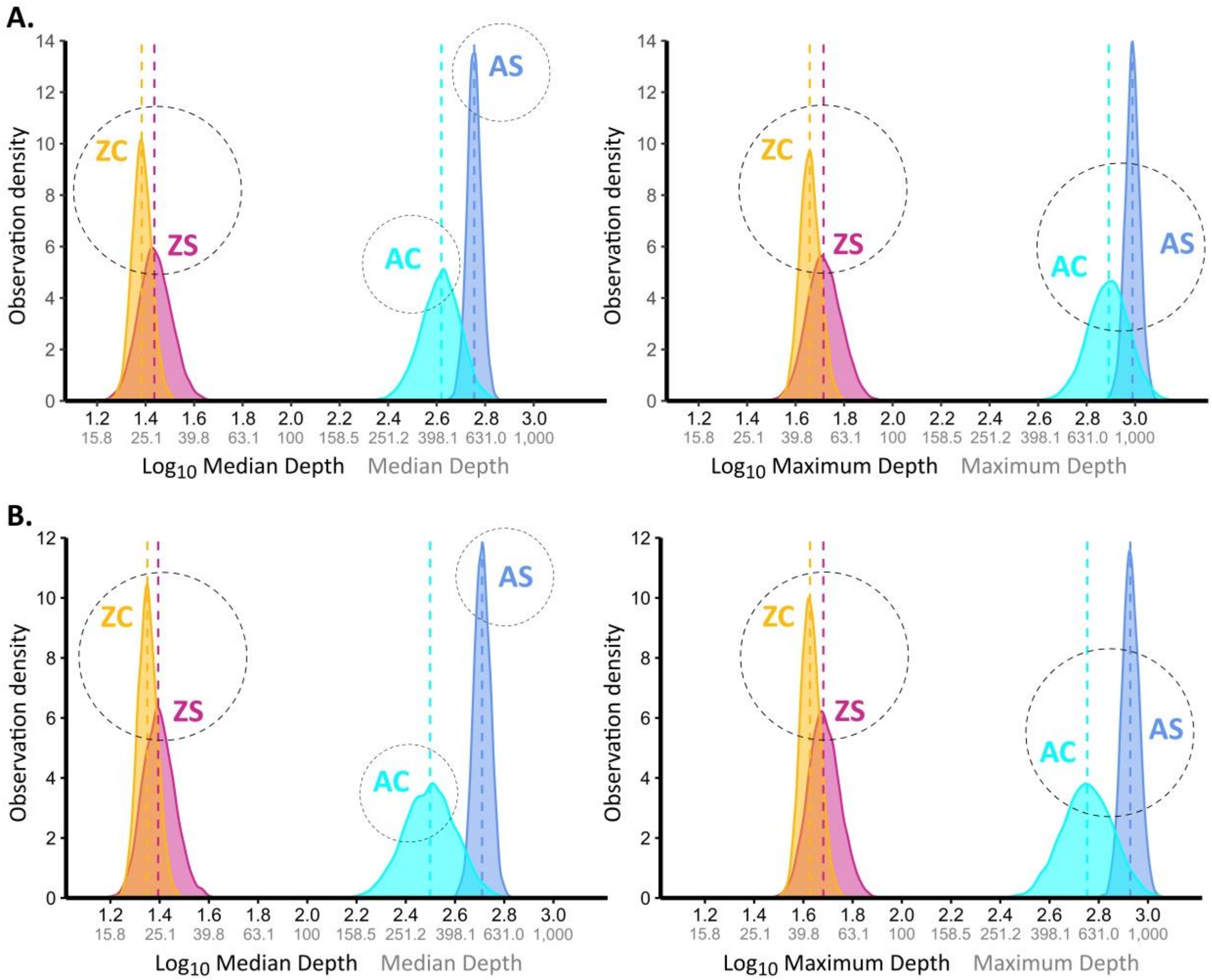

Supplementary Fig. 8 Phylogenetically corrected median and maximum log_{10} depth for four groups of scleractinian corals: AS (azooxanthellate solitary, n_A=107 (n_B=108)), AC (azooxanthellate colonial, $n_{A,B}$=31), ZS (zooxanthellate solitary, $n_{A,B}$=24) and ZC (zooxanthellate colonial, $n_{A,B}$=336), obtained from a variable rate phylogenetic regression model (Supplementary Table 1). Dashed lines point the phylogenetic median of the posterior distribution for the median and maximum depth (median depth: ZC_A=1.38 ~ 24.2 m (ZC_B=1.35 ~ 22.4 m), ZS_A=1.44 ~ 27.3 m (ZS_B=1.39 ~ 24.8 m), AC_A=2.62 ~ 416.0 m (AC_B=2.50 ~ 316.2 m), AS_A=2.75 ~ 568.3 m (AS_B=2.71 ~ 513.0 m); maximum depth: ZC_A=1.66 ~ 45.5 m (ZC_B=1.63 ~ 42.4 m), ZS_A=1.71 ~ 51.9 m (ZS_B=1.68 ~ 48.1 m), AC_A=2.89 ~ 777.8 m (AC_B=2.75 ~ 567.5 m), AS_A=2.99 ~ 975.3 m (AS_B=2.93 ~ 848.3 m)). Dashed circles separate significantly different depths among groups. The significance was assessed as the proportion of the posterior distribution in which differences in estimated coefficients cross zero using a threshold value of 0.05 ($p < 0.05$), i.e., A. median depth: p_{AS-AC}=0.04, p_{AS-ZS}<0.00, p_{AS-ZC}<0.00, p_{AC-ZS}<0.00, p_{AC-ZC}<0.00, p_{ZS-ZC}=0.17; maximum depth: p_{AS-AC}=0.14, p_{AS-ZS}<0.00, p_{AS-ZC}<0.00, p_{AC-ZS}<0.00, p_{AC-ZC}<0.00, p_{ZS-ZC}=0.16; B. median depth: p_{AS-AC}=0.02, p_{AS-ZS}<0.00, p_{AS-ZC}<0.00, p_{AC-ZS}<0.00, p_{AC-ZC}<0.00, p_{ZS-ZC}=0.18; maximum depth: p_{AS-AC}=0.05, p_{AS-ZS}<0.00, p_{AS-ZC}<0.00, p_{AC-ZS}<0.00, p_{AC-ZC}<0.00, p_{ZS-ZC}=0.14. A. Updated database. B. Older database (differences with A. in blue colour).

Supplementary Table 12 Brownian motion (BM) and variable rate (VR) models of depth evolution for four groups of scleractinian corals: AS (azooxanthellate solitary, n=107 (n=108)), AC (azooxanthellate colonial, n=31), ZS (zooxanthellate solitary, n=24) and ZC (zooxanthellate colonial, n=336). Log m Lh: log marginal likelihood. λ: inferred phylogenetic signal - median of the posterior. Log Bayes Factor (BF) higher than 10 indicates very strong evidence in favour of the VR model. Values obtained with the older database highlighted in blue colour.

		BM model		VR model		Log BF
		Log m Lh	λ	Log m Lh	λ	
**Maximum depth**	Run 01	-106.20	0.51	-63.54	0.45	85.32
(log_{10} max. depth)		-107.28	0.37	-73.27	0.45	68.01
	Run 02	-106.21	0.52	-70.61	0.45	71.18
		-107.34	0.37	-73.13	0.45	68.42
	Run 03	-106.21	0.52	-65.35	0.45	81.71
		-107.35	0.37	-73.01	0.45	68.66
**Median depth**	Run 01	-84.00	0.55	-38.19	0.51	91.62
(log_{10} med. depth)		-93.04	0.39	-52.32	0.47	81.44
	Run 02	-84.06	0.55	-38.62	0.40	90.87
		-92.86	0.39	-52.71	0.46	80.30
	Run 03	-84.02	0.55	-38.77	0.52	90.49
		-92.89	0.39	-52.83	0.49	80.13

*Log_{10} depth ~ $\alpha^{AS}+\beta_1^{AC}+\beta_2^{ZS}+\beta_3^{ZC}$

Evolution of depth differences in corals with different traits

Supplementary Table 13 Brownian motion (BM) and variable rate (VR) models of depth evolution for scleractinian corals (n=509 (n=510)). Log m Lh: log marginal likelihood. λ: inferred phylogenetic signal - median of the posterior. Log Bayes Factors (BF) higher than 10 indicates very strong evidence in favour of the VR model. Values obtained with the older database highlighted in blue.

		BM model		VR model		Log BF
		**Log m Lh**	**λ**	**Log m Lh**	**λ**	
**Maximum depth**	Run 01	-218.03	0.87	-172.62	0.88	90.83
(log_{10} max. depth)		-222.14	0.85	-159.23	0.90	125.83
	Run 02	-218.01	0.87	-172.28	0.87	91.46
		-222.11	0.85	-169.32	0.91	105.58
	Run 03	-218.17	0.87	-172.80	0.87	90.75
		-222.21	0.85	-151.74	0.91	140.93
**Median depth**	Run 01	-204.92	0.89	-151.30	0.87	107.25
(log_{10} med. depth)		-214.80	0.86	-137.13	0.90	155.34
	Run 02	-204.69	0.88	-153.92	0.87	101.52
		-214.79	0.86	-154.08	0.89	121.42
	Run 03	-204.99	0.89	-152.92	0.87	104.13
		-214.74	0.86	-140.78	0.90	147.90

Supplementary Table 14 Brownian motion (BM) and variable rate (VR) models of depth evolution, including latitude extension, accounting for differences among four groups of scleractinian corals: AS (azooxanthellate solitary, n=107 (n=108)), AC (azooxanthellate colonial, n=31), ZS (zooxanthellate solitary, n=24) and ZC (zooxanthellate colonial, n=336). Facultative species (n=11) are also included, coded as 0.5 for the two groups they are part of. Log m Lh: log marginal likelihood. λ: inferred phylogenetic signal - median of the posterior. R^2: coefficient of determination - median of the posterior. Log Bayes Factor (BF) higher than 10 indicates very strong evidence in favour of the VR model. Values obtained with the older database highlighted in blue.

		BM model			VR model			Log BF
		**Log m Lh**	**λ**	**R^2**	**Log m Lh**	**λ**	**R^2**	
**Maximum depth**	Run 01	-101.52	0.38	0.55	-68.97	0.39	0.67	65.10
(log_{10} max. depth)		-67.37	0.27	0.56	-67.37	0.35	0.85	100.7
	Run 02	-101.91	0.38	0.55	-67.78	0.39	0.67	68.27
		-67.36	0.26	0.56	-67.36	0.36	0.69	103.01
	Run 03	-101.74	0.38	0.55	-67.72	0.40	0.67	68.02
		-67.67	0.27	0.56	-67.67	0.36	0.69	100.52
**Median depth**	Run 01	-125.29	0.50	0.45	-83.01	0.42	0.66	84.56
(log_{10} med. depth)		-136.97	0.41	0.46	-83.97	0.39	0.64	105.99
	Run 02	-124.33	0.50	0.45	-79.27	0.42	0.66	90.13
		-136.13	0.41	0.46	-86.52	0.38	0.64	99.22
	Run 03	-124.09	0.50	0.45	-83.01	0.42	0.66	82.16
		-135.94	0.42	0.46	-84.20	0.44	0.64	103.49

* Log_{10} depth ~ $\alpha^{AS}+\beta_1^{AS}*(latitude)+\beta_2^{AC}+\beta_3^{AC}*(latitude)+\beta_4^{ZS}+\beta_5^{ZS}*(latitude)+\beta_6^{ZC}+\beta_7^{ZC}*(latitude)$

Supplementary Table 15 Comparison of the model of depth evolution (Supplementary Table 13) and the same model after accounting for the effect of latitudinal extension (Supplementary Table 14). Log m Lh: log marginal likelihood. Log Bayes Factors (BF) higher than 10 indicates very strong evidence in favour of the complex model. Values obtained with the older database highlighted in blue.

		Log m Lh		Log BF
		**Depth**	**Depth ~ Latitude**	
**Maximum depth**	Run 01	-172.62	-68.97	207.3
		-159.23	-67.37	183.72
	Run 02	-172.28	-67.78	209
		-169.32	-67.36	203.92
	Run 03	-172.80	-67.72	210.16
		-151.74	-67.67	168.14
**Median depth**	Run 01	-151.30	-83.01	135.58
		-137.13	-83.97	106.32
	Run 02	-153.92	-79.27	149.3
		-154.08	-86.52	135.12
	Run 03	-152.92	-83.01	139.82
		-140.78	-84.20	113.16
