# Supplementary material for: Deep-sea origin and depth colonization associated with phenotypic innovations in scleractinian corals
Source: Nat Commun. 2023 Nov 17;14:7458. doi: 10.1038/s41467-023-43287-y (PMC10656505; doi:10.1038/s41467-023-43287-y)
Supplement: Supplementary file 3 — Reporting Summary [file 41467_2023_43287_MOESM3_ESM.pdf]

nature portfolio

Corresponding author(s): Ana Navarro Campoy

Last updated by author(s): Oct 31, 2023

Reporting Summary

Nature Portfolio wishes to improve the reproducibility of the work that we publish. This form provides structure for consistency and transparency in reporting. For further information on Nature Portfolio policies, see our Editorial Policies and the Editorial Policy Checklist.

Statistics

For all statistical analyses, confirm that the following items are present in the figure legend, table legend, main text, or Methods section.

n/a	Confirmed	
☐	☒	The exact sample size (*n*) for each experimental group/condition, given as a discrete number and unit of measurement
☐	☒	A statement on whether measurements were taken from distinct samples or whether the same sample was measured repeatedly
☐	☒	The statistical test(s) used AND whether they are one- or two-sided<br>*Only common tests should be described solely by name; describe more complex techniques in the Methods section.*
☐	☒	A description of all covariates tested
☐	☒	A description of any assumptions or corrections, such as tests of normality and adjustment for multiple comparisons
☐	☒	A full description of the statistical parameters including central tendency (e.g. means) or other basic estimates (e.g. regression coefficient) AND variation (e.g. standard deviation) or associated estimates of uncertainty (e.g. confidence intervals)
☐	☒	For null hypothesis testing, the test statistic (e.g. *F*, *t*, *r*) with confidence intervals, effect sizes, degrees of freedom and *P* value noted<br>*Give P values as exact values whenever suitable.*
☐	☒	For Bayesian analysis, information on the choice of priors and Markov chain Monte Carlo settings
☐	☒	For hierarchical and complex designs, identification of the appropriate level for tests and full reporting of outcomes
☐	☒	Estimates of effect sizes (e.g. Cohen's *d*, Pearson's *r*), indicating how they were calculated

Our web collection on statistics for biologists contains articles on many of the points above.

Software and code

Policy information about availability of computer code

Data collection	No software was used.
Data analysis	BayesTraits V3.0 (Baker et al., 2015), available at http://www.evolution.reading.ac.uk/SoftwareMain.html, to perform comparative analyses, and R (R Core Team, 2019) for non-phylogenetic analyses, data manipulation and data visualization. R packages include: phytools, PMCMR, data.table, reader, dplyr, purrr, robustbase, ggplot2 and gridExtra. The R code used to produce the figures, including the Supplementary Information, can be found at https://github.com/anavcampoy/Scleractinia (DOI: 10.5281/zenodo.10053873).

For manuscripts utilizing custom algorithms or software that are central to the research but not yet described in published literature, software must be made available to editors and reviewers. We strongly encourage code deposition in a community repository (e.g. GitHub). See the Nature Portfolio guidelines for submitting code & software for further information.

Data

Policy information about availability of data

All manuscripts must include a data availability statement. This statement should provide the following information, where applicable:

- Accession codes, unique identifiers, or web links for publicly available datasets
- A description of any restrictions on data availability
- For clinical datasets or third party data, please ensure that the statement adheres to our policy

The coral distribution data that support the findings of this study are available at https://github.com/anavcampoy/Scleractinia (DOI: 10.5281/zenodo.10053873)

under the name Database_2019. An updated version of this data is also available as Database_2023. The data to build this database was retrieved from: OBIS (Ocean Biodiversity Information System, https://obis.org, downloaded on 4 April 2019), GBIF (Global Biodiversity Information Facility, https://www.gbif.org, downloaded on 3 April 2019), Corals of the World (http://www.coralsoftheworld.org), CoralTraits (https://coraltraits.org), Worms (https://www.marinespecies.org/), SeaLifeBase (https://www.sealifebase.ca), NOAA (https://www.ncei.noaa.gov/maps/deep-sea-corals/mapSites) and a list of scientific publications.

Research involving human participants, their data, or biological material

Policy information about studies with human participants or human data. See also policy information about sex, gender (identity/presentation), and sexual orientation and race, ethnicity and racism.

Reporting on sex and gender	N/A
Reporting on race, ethnicity, or other socially relevant groupings	N/A
Population characteristics	N/A
Recruitment	N/A
Ethics oversight	N/A

Note that full information on the approval of the study protocol must also be provided in the manuscript.

Field-specific reporting

Please select the one below that is the best fit for your research. If you are not sure, read the appropriate sections before making your selection.

☐ Life sciences ☐ Behavioural & social sciences ☒ Ecological, evolutionary & environmental sciences

For a reference copy of the document with all sections, see nature.com/documents/nr-reporting-summary-flat.pdf

Ecological, evolutionary & environmental sciences study design

All studies must disclose on these points even when the disclosure is negative.

Study description

This study seeks to explain the depth distribution of Scleractinia inferring their origin in the depth gradient and dispersion patterns in terms of directionality and velocity. This inference also takes into account the evolution of two distinct phenotypic traits: symbiosis and coloniality.
A phylogenetic tree with 510 scleractinian corals was used. Depth differences among corals with different traits were evidenced using a phylogenetic ANOVA. The evolution in depth was inferred through phylogenetic bayesian regression models. They model the evolution of the median and maximum species depths separately. The effect of species´ latitude was included with independent intercepts and slopes for each trait-defined group. Brownian motion and Variable rate models of evolution were tested, finding support for rate variation. Using the best resultant model for the median and maximum depths, a phylogenetic tree with scaled branches reflecting evolutionary rates was obtained. This tree was used to test if faster rates have led to species in deeper or shallower waters (directionality trends). This was a bayesian regression model with the median/maximum depth as dependent variable and the branch length as independent variable with intercepts and slopes for each trait-defined group. Then, the ancestral depths were inferred using a phylogenetic predictive model. This model was as the one just described to test trends but including false tips at internal nodes of the tree with scaled branches. In this case, a Generalized Least Squares model was used.
Differences in evolutionary rates among trait-defined groups were also tested through a variable rate model with partitions for each group (n=1,018). The model was run for 500 phylogenetic trees to account for phylogenetic uncertainty. The obtained rates were compared using a Kruskal-Wallis test and post hoc pairwise comparisons using the Wilcoxon rank sum test, adjusting the p-values using the Holm correction.
Depth changes for each trait-defined group were quantitatively evaluated obtaining all ancestor-descendant comparisons and using a two-sided binomial test (n=1,018).
Long-term trends were also assessed using bayesian regression models with depth as independent variable and time of each node/tip as dependent co-variable for each trait-defined group. Two models were effectuated: one for background/slow colonization rates (n=496) and one for fast colonization rates (n=506).
Finally, a bayesian regression model was also used to predict the maximum depth by the rate of evolution for each trait-defined group (n=843).

Research sample

A previously published phylogenetic tree comprising 513 scleractinian corals (Campoy et al. 2020) was used. This sample is a subset of all existing species in the order Scleractinia, including representatives from the two phenotypic traits of interest and encompassing the worldwide distribution of the order. This tree is the most comprehensive for this group and is also dated, enabling us to account for the timing of events that occurred.

Sampling strategy

The sampled species list was restricted by the sampling in the selected phylogenetic tree. Representatives of 32 out of the 33 currently recognized families in the order Scleractinia were included. Three species were pruned out of the tree because rustworthy distribution data for them was not available.

Data collection	ANC collected the data from various data platforms, datasets published in scientific articles, or species descriptions and range expansions. The constructed dataset underwent several quality controls, which are thoroughly described in the manuscript.
Timing and spatial scale	The data was collected at the beginning of this study, from April to September 2019, and it is based on global species distributions. This database was uploaded later, and new data was collected during December 2022 to February 2023.
Data exclusions	The initial dataset comprised species that were present in the phylogenetic tree. However, species without reliable distribution data were directly excluded from the analysis. Additionally, species occurrences were excluded when their accuracy could not be verified by at least two sources. This included isolated points or occurrences at very distant depths from other verified data points.
Reproducibility	All analyses were performed at least three times, and the results consistently supported the findings. These results have been reported either in the main text or the supplementary information.
Randomization	The allocation was not random; however, relevant covariates were included, such as latitude, to account for its effects. Additionally, when possible, analyses were conducted over a sample of phylogenetic trees or random trees taken from a previously published sample to ensure the robustness of our results.
Blinding	Blinding was not necessary for this study as unbiased data collection and analyses were conducted.

Did the study involve field work? ☐ Yes ☒ No

Reporting for specific materials, systems and methods

We require information from authors about some types of materials, experimental systems and methods used in many studies. Here, indicate whether each material, system or method listed is relevant to your study. If you are not sure if a list item applies to your research, read the appropriate section before selecting a response.

Materials & experimental systems

n/a	Involved in the study
☒	☐ Antibodies
☒	☐ Eukaryotic cell lines
☒	☐ Palaeontology and archaeology
☒	☐ Animals and other organisms
☒	☐ Clinical data
☒	☐ Dual use research of concern
☒	☐ Plants

Methods

n/a	Involved in the study
☒	☐ ChIP-seq
☒	☐ Flow cytometry
☒	☐ MRI-based neuroimaging
